# Genomic Features of Open Chromatin Regions (OCRs) in Wild Soybean and Their Effects on Gene Expressions

**DOI:** 10.3390/genes12050640

**Published:** 2021-04-25

**Authors:** Ming-Kun Huang, Ling Zhang, Li-Meng Zhou, Wai-Shing Yung, Man-Wah Li, Hon-Ming Lam

**Affiliations:** 1 School of Life Sciences and Center for Soybean Research of the State Key Laboratory of Agrobiotechnology, The Chinese University of Hong Kong, Shatin, Hong Kong SAR, China; b145331@cuhk.edu.hk (M.-K.H.); zhoulm1993@163.com (L.-M.Z.); vincentyungws@hotmail.com (W.-S.Y.); limanwah@cuhk.edu.hk (M.-W.L.); 2 Guangdong Provincial Key Laboratory for Plant Epigenetics, College of Life Sciences and Oceanography, Shenzhen University, Shenzhen 518055, China; linzh00@126.com; 3 Key Laboratory of Optoelectronic Devices and Systems of Ministry of Education and Guangdong Province, College of Optoelectronic Engineering, Shenzhen University, Shenzhen 518055, China

**Keywords:** wild soybean, open chromatin region (OCR), ATAC-seq, enhancer

## Abstract

Transcription activation is tightly associated with the openness of chromatin, which allows direct contact between transcriptional regulators, such as transcription factors, and their targeted DNA for downstream gene activation. However, the annotation of open chromatin regions (OCRs) in the wild soybean (*Glycine soja*) genome is limited. We performed assay for transposase-accessible chromatin using sequencing (ATAC-seq) and successfully identified 22,333 OCRs in the leaf of W05 (a wild soybean accession). These OCRs were enriched in gene transcription start sites (TSS) and were positively correlated with downstream gene expression. Several known transcription factor (TF)-binding motifs were also enriched at the OCRs. A potential regulatory network was constructed using these transcription factors and the OCR-marked genes. Furthermore, by overlapping the OCR distribution with those of histone modifications from chromatin immunoprecipitation followed by sequencing (ChIP-seq), we found that the distribution of the activation histone mark, H3K4me3, but not that of the repressive H3K27me3 mark, was closely associated with OCRs for gene activation. Several putative enhancer-like distal OCRs were also found to overlap with LincRNA-encoding loci. Moreover, our data suggest that homologous OCRs could potentially influence homologous gene expression. Hence, the duplication of OCRs might be essential for plant genome architecture as well as for regulating gene expression.

## 1. Introduction

During the activation of gene transcription, the binding of transcriptional regulators, such as transcription factors (TFs) or chromatin remodelers, to the chromatin could partially unpack it to form open chromatin regions (OCRs) [1,2,3]. OCRs are usually associated with nucleosome-depleted regions that are usually enriched at the genic region as well as with distal enhancers, which are tightly linked with gene expression [3]. Due to the lack of protection by the nucleosome, the DNA in OCRs is exposed to potential nuclease activities. Thus, high-throughput sequencing of DNA fragments released by digesting the chromatin with externally applied nuclease or sonication has been widely used for genome-wide mapping of OCRs. Established methods include micrococcal nuclease sequencing (MNase-seq) [4,5], DNase I hypersensitivity sites sequencing (DNase-seq) [6], formaldehyde-assisted isolation of regulatory elements sequencing (FAIRE-seq) [7], and assay for transposase-accessible chromatin using sequencing (ATAC-seq) [3,8]. Among these technologies, ATAC-seq is one of the most convenient methods for OCR identification. Making use of the hyperactive Tn5 mutant [9,10], ATAC-seq requires significantly lower amounts of the starting material as well as shorter library construction time compared to other methods. According to the Encyclopedia of DNA Elements (ENCODE) project [11], ATAC-seq has been widely applied in mammalian genomic studies to identify more than tens of thousands of OCRs, which are essential for analyzing mammalian genome architecture. Due to the regulatory importance of OCRs, the application of ATAC-seq for mapping OCRs has been reported in the model plant Arabidopsis [1,8,12], maize [13,14,15], and other plants [16,17].

Soybean is one of the essential crops grown worldwide. Compared to the cultivated soybean (*Glycine max*), wild soybean (*Glycine soja*) germplasms have distinct agronomic traits, which serve as potential genetic resources for quality improvement in soybean breeding [18]. The availability of a high-quality reference genome of wild soybean [18] allows precision charting of OCRs. However, unlike cultivated soybean and other crops, such as maize and rice, the OCRs in wild soybean are less studied and remain elusive. Here, in this study, we identified the OCRs in the leaf of the wild soybean accession, W05, via ATAC-seq. The results showed that these regions were associated with the expression of the nearest genes, and they were enriched with TF-binding motifs. The OCRs marked with distinct histone modifications were also identified via comparing the ATAC-seq data with the histone modification profiles generated from chromatin immunoprecipitation followed by sequencing (ChIP-seq). OCR duplications associated with homologous protein-coding genes were also observed in this study. In contrast, the loss of the OCR in one gene within the homologous pair appeared to affect its expression. Together, these results reveal the potential regulatory role of OCRs in wild soybean, and the data serve as genomic resources for further studies on whether the variations in noncoding regulatory OCRs across different germplasms would affect soybean domestication.

## 2. Materials and Methods

### 2.1. Plant Materials

Wild soybean germplasm, W05, was used in this study [18]. Soybean seeds were scarred and then germinated in vermiculite for 7 d and then transferred to half-strength Hoagland’s solution [19] in a greenhouse under natural light and temperature until the first trifoliate leaves were fully developed for sample collection.

### 2.2. RNA Sequencing (RNA-Seq) Data Analysis

The RNA-seq data of soybean trifoliate leaves with the accession numbers SRR8552880, SRR8552881, and SRR8552883 were downloaded from NCBI [18]. Adaptors in the raw reads were trimmed using TrimGalore (http://github.com/FelixKrueger/TrimGalore, accessed on 5 September 2020, default parameters). The filtered reads were mapped to the W05 reference genome [18], and the normalized gene expression levels and fragments per kilobase per million mapped reads (FPKM) were calculated by the HISAT-StringTie pipeline with default parameters [20].

### 2.3. Assay for Transposase-Accessible Chromatin Using Sequencing (ATAC-Seq)

The ATAC-seq library preparation was performed according to a previous study [17]. In brief, about 0.2 g of W05 trifoliate leaves was ground into powder in liquid N_2_. The nuclei were extracted with 10 mL lysis buffer (10 mM Tris-HCl, pH 8.0, 0.25 M sucrose, 0.5% Triton X-100, 1 X Protease Inhibitor [PI]) and washed with 1 mL wash buffer I (10 mM Tris-HCl, pH 8.0, 0.5% Triton X-100, 1 X PI) and wash buffer II (10 mM Tris-HCl, pH 8.0, 5 mM MgCl_2_). Around 10^5^ nuclei were resuspended in a 50 μL tagmentation reaction of the TruePrep DNA Library Prep Kit V2 for Illumina (Vazyme, TD501, Nanjing, China) and incubated at 37 °C for 30 min. The tagged DNA was purified with the DNA Clean and Concentrator Kit (Zymo Research, D4014, Irvine, CA, USA) and amplified with Q5 DNA polymerase (NEB, M0491) and the indexed primers from TruePrep Index Kit V2 for Illumina (Vazyme, TD202, Nanjing, China). The ATAC-seq libraries were sequenced on the Illumina X Ten platform in PE150 mode. The adaptors of ATAC-seq raw data were trimmed using the TrimGalore software, and the filtered reads were mapped to the W05 reference genome using Bowtie2 [21]. The mapped reads (MAPQ > 30) were used to identify the enriched regions via Genrich (https://github.com/jsh58/Genrich, accessed on 4 August 2019) with the ATAC model (-j).

### 2.4. Motif Analysis

The MEME suite software package [22] was used for motif analyses using the default parameters. The transcription factor (TF) motif position weight matrix (PWM) was downloaded from PlANTPAN3.0 [23].

### 2.5. Chromatin Immunoprecipitation-Sequencing (ChIP-Seq)

ChIP-seq of trifoliate leaves of W05 was performed following the procedures of a previous study with minor modifications [24]. ChIP-seq grade anti-H3K27me3 (Diagenode, C15410195, Denville, NJ, USA) and anti-H3K4me3 (Millipore, 07473, Burlington, VT, USA) antibodies were used for chromatin immunoprecipitation. About 5 ng ChIP’ed DNA or input DNA was used for ChIP-seq library construction via TruePrep DNA Library Prep Kit V2 for Illumina (Vazyme, TD501, Nanjing, China). ChIP-seq libraries were sequenced in PE150 mode of the Illumina X Ten platform. Adaptor trimming, low-quality reads removing, and mapping were performed using TrimGalore and Bowtie2. The mapped reads (>MAPQ30) were used for peak-calling using MACS2 [25], and the parameters were set as ‘—trackline —extsize 147 —broad -q 0.01 —nomodel -g 1.0e + 9 —buffer-size 500,000’. An input library was used as control.

## 3. Results

### 3.1. Identification of OCRs by ATAC-Seq in W05 Leaf

**Figure 1:**
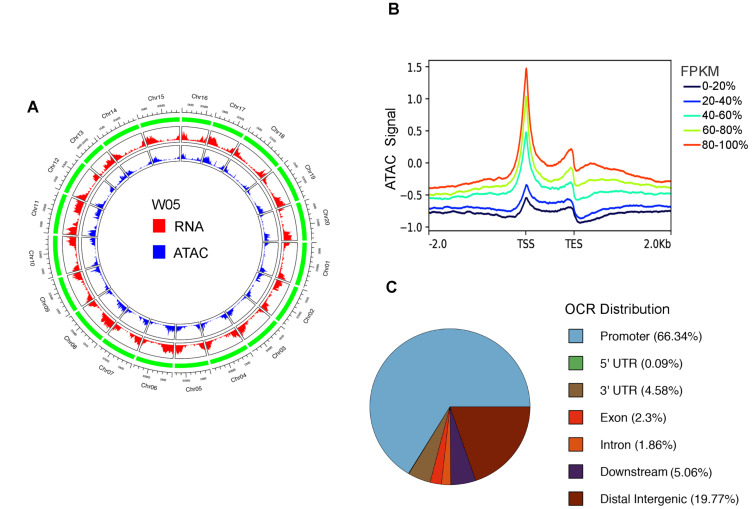
Data summary of the assay for transposase-accessible chromatin using sequencing (ATAC-seq) on the leaf of W05 (wild soybean). (**A**) Overview of the coverage of W05 leaf RNA-seq and ATAC-seq in 20 chromosomes. (**B**) W05 leaf ATAC-seq coverage is positively correlated to the gene expression level. The color range from black to red indicates the increasing gene expression level (sorted by FPKM). TSS, transcription start site; TES, transcription end site. (**C**) Distribution of OCRs in the W05 genome.

We adopted ATAC-seq for genome-wide characterization of OCRs in the wild soybean accession W05. In total, two biological replicates of leaf ATAC-seq libraries generated about 40.8 and 30.0 million (M) filtered reads, respectively (Table S1). We then mapped the reads to the W05 reference genome [18]. As expected, we found that the ATAC-seq signals were mainly enriched at the gene transcription start site (TSS) and the signal intensities were positively correlated with the expression levels of the tagged genes, suggesting that the open chromatin status is associated with gene activation (Figure 1A,B). Furthermore, 22,333 enriched regions in the ATAC-seq were identified using the Genrich software (see method) and defined as OCRs (Table S2). This number is similar to the number of OCRs identified in cultivated soybean leaves [17]. Furthermore, these OCRs were associated with 15,588 protein-coding genes in total (Table S2). Moreover, to describe the OCR distribution in the W05 genome, we annotated these OCRs according to the W05 genomic features. The results showed that most of the OCRs were located in the promoter (66.34%) and distal intergenic (19.77%) regions, with the rest belonging to the downstream (5.06%), 3′ untranslated region (UTR, 4.58%), exon (2.3%), intron (1.86%), and 5′ UTR (0.09%) of a genic region (Figure 1C and Table S2).

### 3.2. Transcription Factor-Binding Motif Enrichment in the OCRs and Their Potential Roles in Gene Regulation

**Figure 2:**
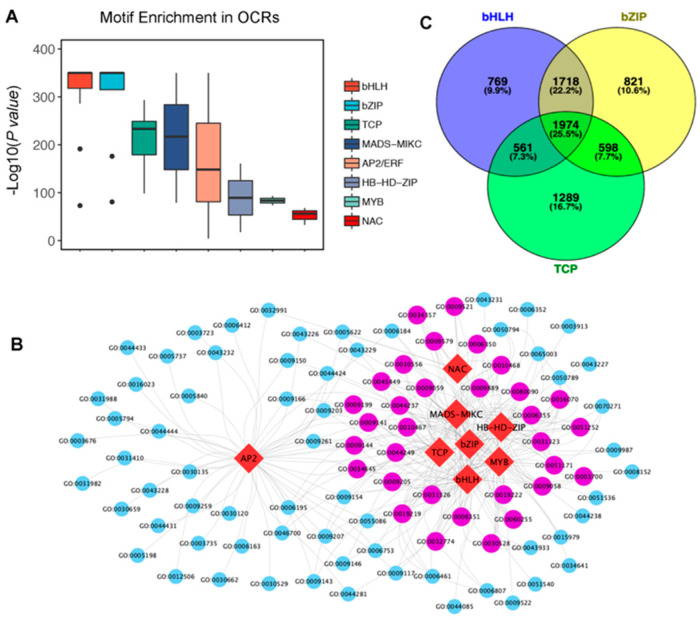
Conserved transcription factor (TF) motifs enriched at open chromatin regions (OCRs).(**A**) Top eight enriched TF motifs detected in the OCRs, including the bHLH, bZIP, TCP, MADS-MIKC, AP2/ERF, HB-HD-ZIP, MYB, and NAC motifs. (**B**) A potential regulatory network of genes based on TF motif–OCR associations. Purple circle indicates a Gene Ontology (GO) term targeted by at least four TF motifs in OCRs, while blue circle indicates a GO term linked to fewer than three TF motifs. Red rhombus represents a TF motif. (**C**) A Venn diagram showing potential cotargeted genes by the bHLH, bZIP, and TCP families of TFs, the top three most significantly enriched TF motifs in the OCRs.

Relaxing of the chromatin structure enables the binding of transcription factors (TFs) to activate gene expression, and thus OCRs usually harbor conserved TF-binding motifs [26]. To assess the enrichment of TF-binding motifs at these OCRs, we mapped the known TF-binding motifs listed in the PlantPAN3.0 database [23] to these OCRs. A significant enrichment of 48.9% (70 out of 143) of the conserved TF-binding motifs was observed at the OCRs in our database (Tables S3 and S4), among which bHLH, bZIP, TCP, MADS, AP2/ERF, HD-ZIP, MYB, and NAC were the eight most significantly enriched motifs (Figure 2A). Moreover, using the enriched TF-binding motifs as well as the Gene Ontology (GO) terms of the OCR-associated genes downstream, we constructed a potential TF regulatory network in the W05 leaf. Seven TF families formed a relatively close network (Figure 2B, Table S5). About 33 GO terms (Figure 2B, purple circle) were tightly coregulated by these seven TF families. On the other hand, the AP2/ERF family regulated a downstream subset that showed less overlapping with the seven TF families mentioned above (Figure 2B, blue circle), indicating that AP2/ERF might regulate a unique set of pathways. Furthermore, 1974 genes (25.5% of all OCR-associated genes) were found to be cotargeted by the top three enriched motifs from the bHLH, bZIP, and TCP families. These results suggest that multiple TFs may cooperate in the OCRs to regulate downstream gene activation.

### 3.3. Histone Modifications in OCRs and Their Roles in Gene Activation and as Putative Enhancers

**Figure 3:**
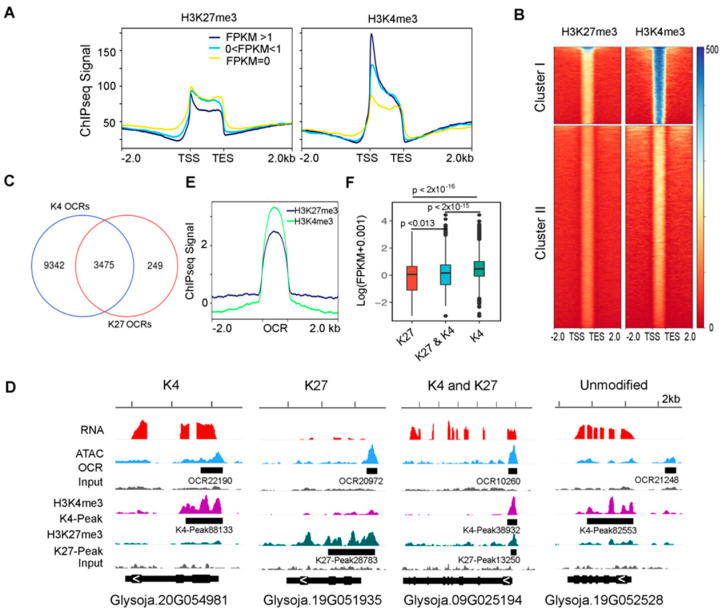
Histone modifications at OCRs. (**A**) Enriched H3K27me3 (histone H3 lysine 27 trimethylation) and H3K4me3 (histone H3 lysine 4 trimethylation) ChIP signals were negatively and positively correlated with gene expressions, respectively. TSS, transcription start site; TES, transcription end site. (**B**) A heatmap showing the OCRs classified into two major clusters according to the H3K27me3 and H3K4me3 signal intensities using K-mean algorithm. TSS, transcription start site; TES, transcription end site. (**C**) A Venn diagram showing the number of OCRs that were modified by either H3K4me3 or H3K27me3 and those that were modified by both. (**D**) Examples of the H3K4me3 (K4)- and H3K27me3 (K27)-modified, K4 and K27 dual-modified, and unmodified OCRs. Black boxes indicate the identified OCRs or histone modification peaks. (**E**) H4K4me3 marks had more signal coverage than H3K27me3 marks at K4 and K27 dual-modified OCRs. (**F**) The expression levels of genes associated with K4-modified OCRs were significantly higher than those with K27-modified or K4 and K27 dual-modified OCRs. The *p* value was calculated by Wilcoxon test.

As shown in previous studies, OCRs could be associated with gene-activating or gene-repressing histone marks [15,16]. Since H3K27me3 (histone H3 lysine 27 trimethylation) and H3K4me3 (histone H3 lysine 4 trimethylation) have been widely used to represent gene repression or activation marks in plants [16], we performed ChIP-seq on H3K27me3 and H3K4me3 using W05 leaves to investigate the relationship between their distributions and OCRs. In total, we identified 31,346 H3K27me3- (Table S6) and 88,283 H3K4me3- (Table S7) enriched peaks. As expected, the H3K27me3 signals were negatively correlated with gene expressions, while H3K4me3 signals showed a positive correlation with gene expressions (Figure 3A), following a similar pattern to the ATAC-seq signal. Furthermore, using the K-mean algorithm, the OCRs could be classified into two major clusters (Figure 3B). Cluster-II OCRs were associated with little H3K27me3 or H3K4me3 signals and were therefore termed unmodified OCRs. Most of OCRs in Cluster I were modified by H3K4me3 and some, to a lesser extent, by H3K27me3. There were 9342 OCRs marked with H3K4me3 only (K4 OCRs) and just 249 OCRs were marked with H3K27me3 only (K27 OCRs) (Figure 3C,D). Although there were 3475 OCRs modified by both H3K4me3 and H3K27me3, the overall H3K4me3 signal coverage was higher than that of H3K27me3 in these regions (Figure 3E, Table S8). Moreover, genes associated with K4 OCRs had higher expression than those associated with K27 OCRs or K4 and K27 dual-modified OCRs (Figure 3F, Table S8). Taken together, the activation histone modifications such as H3K4me3, rather than the repressive H3K27me3, were tightly correlated with chromatin openness.

**Figure 4:**
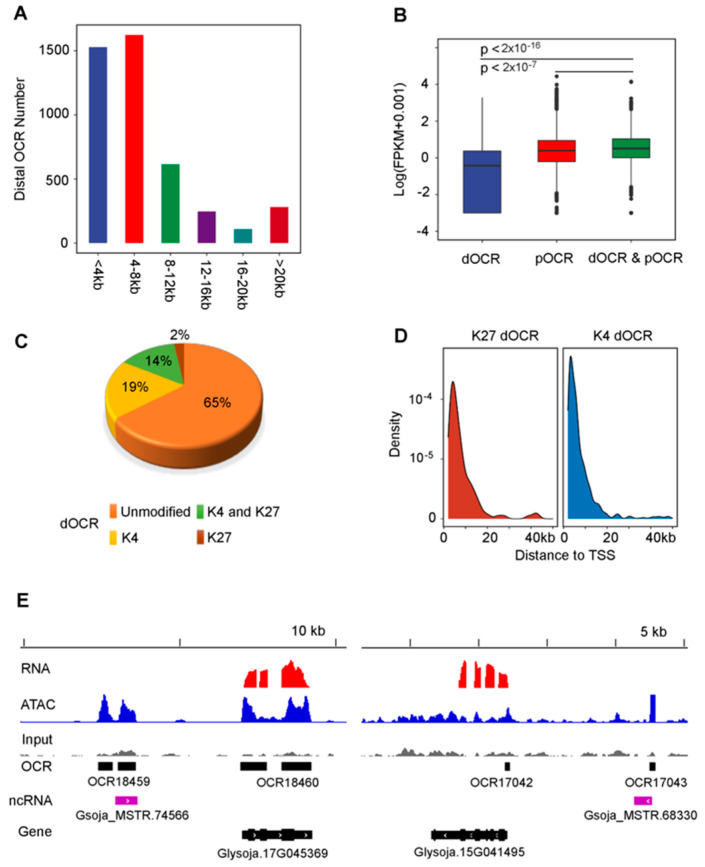
The genomic features of distal OCRs (dOCRs). (**A**) A histogram of the distances from dOCRs to the transcription start site (TSS) of their nearest associated genes. (**B**) The expression levels of genes associated with both promoter OCR (pOCR) and dOCRs (in green) were significantly higher than those with only dOCR (in blue) or pOCR (in red). The *p* value was calculated by Wilcoxon test. (**C**) The percentages of unmodified, K4-modified, K27-modified, and K4 and K27 dual-modified dOCRs. K4 and K27 here refer to the trimethylated lysine residues 4 and 27 on histone H3, respectively. (**D**) The distribution of K4- and K27-modified dOCRs according to their distance to TSS. TSS, transcription start site. (**E**) Examples of dOCRs that overlap with long intergenic noncoding RNAs (LincRNAs), which are putative enhancers.

Distal OCR (dOCR) is another important category of OCRs because it is widely used for the prediction of putative enhancers in plants [14,27,28]. In this study, we found 4405 OCRs located upstream of the 2-kb promoter region. We defined these OCRs as dOCRs, 1529 and 1624 of which were located at the 2–4 and 4–8 kb regions, respectively, upstream from the transcription start site (TSS) of the nearest gene. The longest distance between the dOCR (W05_OCR09684) and the TSS of the nearest gene was as much as 300 kb (Figure 4A, Table S2). It was observed that genes associated with both dOCRs and promoter OCRs (pOCRs) tended to have higher expressions than those only associated with either dOCRs or pOCRs (Figure 4B), indicating that these dOCRs might serve as putative enhancers for gene expression. Compared to pOCRs, most of the dOCRs (65%) had fewer H3K4me3 or H3K27me3 modifications (Figure 4C, Table S8). The distribution of H3K4 and H3K27 histone modifications of dOCRs within the 10 kb region upstream from TSS (Figure 4D) indicated that the chromatin state of dOCRs increasingly shifted to the unmodified state with increasing distance from TSS, similar to what has been observed in other plant species [16]. In addition, enhancers are sometimes associated with loci that code for long intergenic noncoding RNAs (LincRNAs), which are often considered to be transcriptional enhancer RNAs (eRNAs) [2,28]. Similar to the cultivated soybean, a small proportion (3.6%) of dOCRs in this study overlapped with the previously reported LincRNA loci in W05 [29] (Table S9), suggesting that these dOCRs could be putative enhancers.

### 3.4. OCR Duplication Has Potential Effects on the Expressions of Homologous Genes

**Figure 5:**
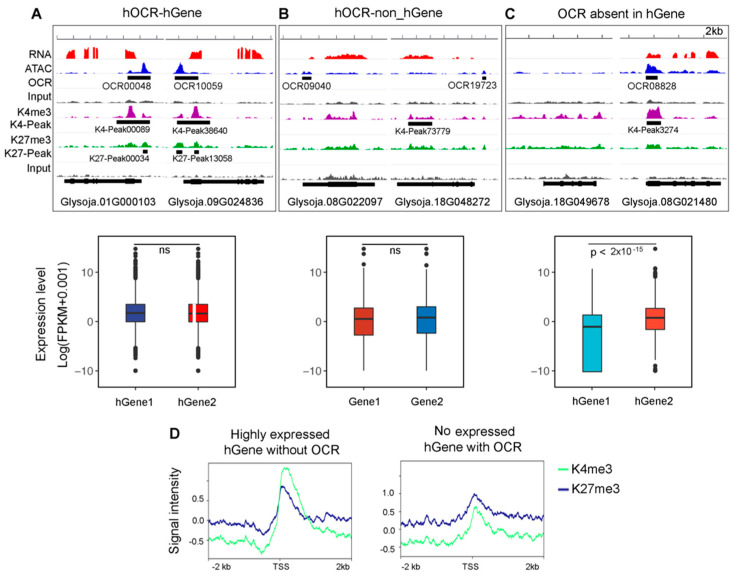
Homologous OCRs (hOCRs) can potentially influence homologous gene (hGene) expressions. (**A**) A representative case of two hGenes targeted by hOCRs (upper panel) and the comparison of expression levels between the two hGenes (lower panel). (**B**) A representative case of two non-hGenes targeted by hOCRs (upper panel) and the comparison of expression levels between the two non-hGenes (lower panel). (**C**) A representative case of two hGenes without hOCRs (upper panel) and the comparison of expression levels between these two hGenes (lower panel). K4me3 (trimethylated lysine 4) and K27me3 (trimethylated lysine 27) here refer to modifications on histone H3 specifically. ns, not significant. The *p* value was calculated by the Wilcoxon test. (**D**) Histone modifications antagonized the effect of OCR on hGene expression. Genes with FPKM more than 10 were defined as highly expressed genes and those with FPKM equal to 0 as nonexpressed gene.

As a paleopolyploid plant, soybean has undergone two rounds of whole-genome duplication (WGD) recently, and some have suggested that the diploidization of the soybean genome has not yet been completed [30], during which an extensive amount of protein-coding genes are duplicated and retained in the genome. Therefore, we wonder whether OCRs underwent the same duplication process similar to the protein-coding genes. The results showed that a homologous OCR (hOCR), with high sequence similarity (E-value < 1 × 10^−5^1e^−5^), could be found on another chromosome for 40.3% of the OCRs (Table S10). This proportion is similar to that of the duplicated coding genes [30]. As was expected, pOCRs tended to have their homologous counterparts in the promoter regions and dOCRs in the distal regions (Table S10). Furthermore, most of the hOCRs (80%) were linked to homologous genes (hGenes), indicating that the hOCR–hGene associations were duplicated together during the WGD events (Figure 5A, upper panel). Furthermore, the overall expressions of these hGenes associated with hOCRs did not show significant differences between the homologous pair (Figure 5A, lower panel). Interestingly, about 20% of the hOCRs were associated with non-hGenes (Figure 5B, upper panel), and the overall expressions of the non-hGenes showed similar expression levels as those of the hGenes (Figure 5B, lower panel). Furthermore, we found 5879 hGene pairs in which only one member in each homologous pair was associated with OCR (Figure 5C, upper panel, Table S11). The overall expressions were significantly higher for the hGenes with an OCR compared to their homologous counterparts without an OCR (Figure 5C, lower panel), indicating that the absence of the OCR could affect the hGene expression. These results partially support that hGene expression levels may be dependent on the duplicated hOCRs. In addition, among these genes pairs, we observed that a subset of hGenes without association with OCR displayed high expression (FPKM > 10) and another subset of hGenes with OCR were not expressed (FPKM = 0). We further examined the histone modifications at the TSS region of these hGenes. Higher enrichment level of activation histone marks (K4me3) than the repressive marks (K27me3) were observed at the TSS of highly expressed hGenes without OCR (Figure 5D, left panel). In contrast, the undetectable expression of hGenes equipped with OCR might be the result of highly enriched level of K27me3 at TSS (Figure 5D, right panel). Taken together, the antagonization of activation and respressive histone modifications would be another important factor affecting the regulatory role of hOCRs on gene expression.

## 4. Discussion

In this study, we adopted ATAC-seq for genome-wide identification of OCRs in a wild soybean. As indicated by the RNA-seq data, OCRs were positively associated with gene expression. Moreover, conserved motifs of TF families were found to be enriched in these OCRs, indicating that these regions are important for gene activation. Furthermore, OCRs were more likely to couple with the active histone mark (H3K4me3) rather than the repressive mark (H3K27me3) in regulating transcription. Finally, the absence of hOCRs might affect the expression level of hGenes. Together, this genome-wide profile of OCRs could serve as a potential resource for further investigation of the mechanisms of gene regulation in soybean.

Currently, OCR profiling using ATAC-seq in plants has been reported, including in Arabidopsis [1,12], maize [15,16,31], and cultivated soybean [16,17], but less so in wild soybean [32]. In this study, the 22,333 OCRs we identified in wild soybean is similar to the OCR number previously reported in cultivated soybean [16,17]. Moreover, the association with histone marks as well as the distal enhancer-like feature of dOCRs are consistent with the published OCR profiles in other plants [16], suggesting that the genomic features of OCRs are conserved among plants.

OCRs usually harbor conserved cis-regulatory elements, such as TF-binding motifs, in either the promoter or the distal region. In this study, we observed that there were several significantly enriched TF-binding motifs in the OCRs and that the absence of hOCRs could differentiate the expression levels between members of a pair of hGenes. This raises an interesting question of whether these noncoding DNA regions have also contributed to the adaptations, divergence, and domestication of soybean, which is worth further investigation. Variations in noncoding regions have been reported as important resources for crop domestication in maize [33] and rice [34]. In maize, the increased apical dominance of the maize TEOSINTE BRANCHED 1 (tb1) mutant is due to a transposon insertion into the maize distal enhancer within the dOCR. Further studies have reported that this dOCR might be under the control of ARF transcription factors [31,33]. Indeed, with the rapid development of CRISPR/Cas9 technology, it is now possible to apply genome editing to noncoding cis-elements [35]. In a recent report, cis-regulatory mutagenesis via CRISPR/Cas9 in a plant homeobox gene uncovered the pleiotropy in plant architecture [36]. Quantitative trait locus (QTL) mapping studies or genome-wide association studies (GWASs) usually focus on the coding genes for elucidating causal polymorphisms. Previous studies have demonstrated that the less-explored noncoding elements could also possess the causal polymorphisms of the target phenotypes. Given that the OCR profiles in both cultivated and wild soybean are currently available, these genomic regions can now be used in GWASs to identify potential noncoding loci, which are essential for the soybean genome architecture.

## 5. Conclusions

Using ATAC-seq, we successfully identified the open chromatin regions (OCRs) in the trifoliate leaf of a wild soybean. TF motif enrichment analysis in combination with the histone mark profiles suggest that OCRs are important for promoting gene expression. The expression bias between hGene members with and without hOCRs indicate that OCRs may play important roles during whole-genome duplication in soybean and subsequent subfunctionalization of duplicated genes. Together, these data serve as important resources for further genomic investigations in wild soybean.

## Data Availability

The raw data in this study have been submitted to the National Center for Biotechnology Information (NCBI) under the BioProject accession PRJNA716521.

## Supplementary Materials

The following are available online at https://www.mdpi.com/article/10.3390/genes12050640/s1, Table S1: The list of raw data from ATAC-seq, ChIP-seq, and RNA-seq in this study. Table S2: The genomic annotation of W05 OCRs. Table S3: The TF motif position weight matrix (PWM) used in this study. Table S4: TF motif enrichment analysis. Table S5: Gene Ontology (GO) terms potentially targeted by the specific TF families. Table S6: The genomic annotation of H3K27me3-enriched peaks in the leaf of W05. Table S7: The genomic annotation of H3K4me3-enriched peaks in the leaf of W05. Table S8: The histone modification status of OCRs. Table S9: The overlapping between dOCRs and LincRNAs. Table S10: List of homologous OCRs (hOCRs) found in this study. Table S11: Expression levels of homologous genes (hGenes) without hOCRs.

## Sample Availability

Not available.
